# Sea Anemones: Quiet Achievers in the Field of Peptide Toxins

**DOI:** 10.3390/toxins10010036

**Published:** 2018-01-08

**Authors:** Peter J. Prentis, Ana Pavasovic, Raymond S. Norton

**Affiliations:** 1 School of Earth, Environmental and Biological Sciences, Queensland University of Technology (QUT), Brisbane, QLD 4001, Australia; p.prentis@qut.edu.au (P.J.P.); a.pavasovic@qut.edu.au (A.P.); 2 Institute of Future Environments, Queensland University of Technology (QUT), Brisbane, QLD 4001, Australia; 3 Faculty of Health, Queensland University of Technology (QUT), Brisbane, QLD 4001, Australia; 4 Medicinal Chemistry, Monash Institute of Pharmaceutical Sciences, Monash University, Parkville, VIC 3052, Australia

**Keywords:** sea anemone, peptide, ShK, potassium channel, autoimmune disease, genomics, transcriptomics, proteomics, evolution, The sea anemone peptide ShK highlights the potential of these venomous animals to produce valuable therapeutic leads, and the abundance in nature of peptides related to ShK suggests that this scaffold can support a range of functions. Current genomic, transcriptomic and proteomic studies of sea anemones promise to greatly expand the number of ShK analogues and to identify a range of novel peptide families.

## Abstract

Sea anemones have been understudied as a source of peptide and protein toxins, with relatively few examined as a source of new pharmacological tools or therapeutic leads. This is surprising given the success of some anemone peptides that have been tested, such as the potassium channel blocker from *Stichodactyla helianthus* known as ShK. An analogue of this peptide, ShK-186, which is now known as dalazatide, has successfully completed Phase 1 clinical trials and is about to enter Phase 2 trials for the treatment of autoimmune diseases. One of the impediments to the exploitation of sea anemone toxins in the pharmaceutical industry has been the difficulty associated with their high-throughput discovery and isolation. Recent developments in multiple ‘omic’ technologies, including genomics, transcriptomics and proteomics, coupled with advanced bioinformatics, have opened the way for large-scale discovery of novel sea anemone toxins from a range of species. Many of these toxins will be useful pharmacological tools and some will hopefully prove to be valuable therapeutic leads.

## 1. Introduction

**Figure 1:**
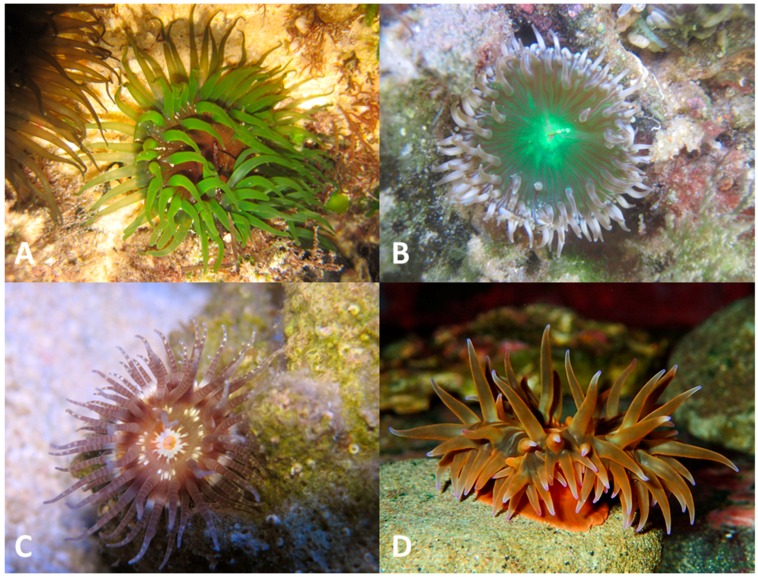
Sea anemone species that are locally abundant in the waters off south-eastern Queensland, Australia; these species have been largely unexplored for their toxin contents and are currently under investigation in our labs to identify new peptide and protein toxins with potentially novel functions. (**A**) *Aulactinia veratra* (**B**) undescribed species of *Anthopleura* (**C**) *Calliactis polypus* (**D**) *Actinia australiensis*. Photo credit: Ana Pavasovic.

Sea anemones are members of the phylum Cnidaria, class Anthozoa, subclass Hexacorallia and order Actiniaria, one of the oldest extant orders of venomous animals. In common with many other venomous animals, they produce venom that is a complex mixture of small molecules, peptides and proteins [1,2,3,4,5,6,7]. Unlike some of the better known groups of venomous animals such as snakes and spiders, however, or even their fellow cnidarians the Australian box jellyfish or Irukandji, which (quite appropriately) attract considerable public attention [8,9], the potentially harmful consequences of contact with sea anemones are relatively unknown. Indeed, many members of the public are not even aware that they are animals rather than plants, let alone venomous ones (Figure 1).

While most injuries caused by sea anemones are associated with skin rashes and oedema, more extreme reactions have been reported for several species, including *Actinodendron plumosum* and other species from the family Actinodendronidae (species from this family are collectively known as the hell’s fire anemones), *Telmatactis* species, *Phyllodiscus semoni* (night or wasp anemone), *Actinia equina* (beadlet anemone) and *Anemonia sulcata* (the snakelocks anemone, synonomy *Anemonia viridis*) [10,11]. For example, a swimmer lost consciousness and underwent cardiopulmonary arrest after being stung by the sea anemone, *Actinia equina*, although this may have been a consequence of an anaphylactic reaction following prior exposure to unknown sea anemones [12]. Sea anemones from the family Aliciideae are known to be particularly dangerous to humans, with severe reactions observed following contact with both *Triactis producta* [13] and *P. semoni* [10]. In fact, *P. semoni* is responsible for one of the few fatalities to result from sea anemone envenomation [14]. The venom from *P. semoni* has caused acute renal failure in humans, with a protein toxin (PsTX-115) from this venom causing severe kidney damage in rat models [15].

## 2. Venom Apparatus

**Figure 2:**
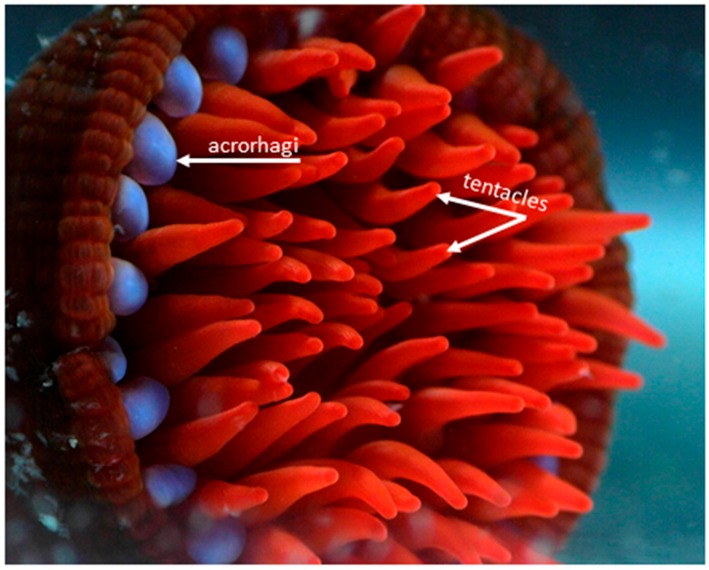
This photograph shows the bright blue acrorhagi used in intraspecific combat [20] and red tentacles used in prey capture and defence against predators of the Australian sea anemone, *Actinia tenebrosa*. Both structures have a high density of nematocysts, but they have different nematocysts complements (for example, holotrichs are abundant in acrorhagi and basitrichs in tentacles). Photo credit: Chloe van der Burg.

**Figure 3:**
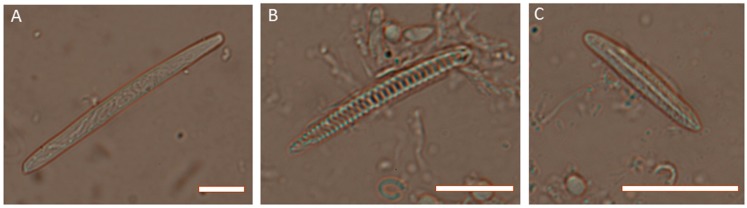
Photomicrographs of cnidae from *Actinia tenebrosa*. (**A**) holotrich from acrorhagi; (**B**) spirocyst from tentacles; (**C**) basitrich from tentacles. Scale bars are 5 µm in each case. Photo credit: Michela Mitchell.

Sea anemones, in common with other members of the phylum Cnidaria, possess numerous specialized stinging cells (cnidocytes) that are widely distributed throughout the body [16]. These stinging cells are equipped with organelles known as nematocysts (cnidae), which contain small threads that are forcefully everted when stimulated mechanically or chemically [17]. These nematocysts contain a complex cocktail of toxins that is used to envenomate predatory and prey species upon discharge [1,3,6]. Nematocysts show significant heterogeneity in their density and morphology across different structures within sea anemones [18]. For example, in *Actinia tenebrosa* (or its northern hemisphere relatives *Actinia equina* and *Actinia fragacea*), acrorhagi (which are used in aggressive intra-specific combat (Figure 2)) contain holotrichs and basitrichs [19] (Figure 3), while the tentacles (which are used in prey capture and defence) contain basitrichs and spirocysts; basitrichs are also found in the mesenteric filaments, column, pedal disc and actinopharynx [19] (Figure 3). The differences in cnidae composition are related to differences in the functional specialization of morphological structures, i.e., the capture of prey (crustaceans, small fish), defence against predators and intraspecific aggression [19,20,21,22]. 

## 3. Peptide Toxins from Anemones

While many families of sea anemone toxins have been described already [7,23], much still remains to be discovered about toxins in this group. In fact, there are only 236 peptide or protein toxins in the manually curated ToxProt database [24], which have been isolated from just 45 sea anemone species. This means that fewer than four percent (45 of more than 1100 species) of all sea anemones have had their venom peptides and proteins examined. Currently, anemone toxins can be classified into 15 known families (Table 1), but 19 toxin peptides and proteins remain largely uncharacterized. The four most common toxin protein families isolated from anemones are the actinoporin family, sea anemone subfamily (30 proteins found in the ToxProt database); sea anemone sodium channel inhibitory toxin family, type I subfamily (52 proteins found in the ToxProt database); sea anemone type 3 (BDS) potassium channel toxin family (32 proteins found in the ToxProt database), and venom Kunitz-type family, sea anemone type 2 potassium channel toxin subfamily (26 proteins found in the ToxProt database). Of these four toxin protein families, only the actinoporin family, sea anemone subfamily, has been found outside the Actinioidea superfamily of anemones based on sequences currently available in the ToxProt database. In fact, 206 of the 236 toxin proteins in the ToxProt database are from the Actinioidea superfamily. This indicates that the species that have been examined show a strong taxonomic bias (Table 1), and we therefore need to examine the venom peptide profile from sea anemone species from other superfamilies. Consequently, there is every reason for optimism that the remaining 96% of species, particularly those distantly related to the superfamily Actinioidea, will provide interesting new peptides, some of which will no doubt have therapeutic potential. Some of the more venomous species, such as *Telmatactis australiensis*, *Dofleinia armata* and *Triactis producta*, should be especially interesting in this context.

This article will focus mainly on ShK domains (which, in sea anemones, are members of the type 1 potassium channel toxin family, Type 1b subfamily, Table 1) because of their demonstrated therapeutic potential and broad distribution in nature, as documented in the next section. However, several other classes of peptide toxins from sea anemones have been investigated as therapeutic leads or pharmacological tools. For example, the sodium channel toxins anthopleurin-A and -B showed initial promise as positive inotropes for use in cardiovascular disease [25,26], although they and their homologues from other anemones, for example ATX-I and -II and ShI [27,28], have also proven to be valuable probes of site 3 on voltage-gated sodium channels [29], which mediates channel inactivation. APETx2, from *Anthopleura elegantissima*, inhibits the acid-sensing ion channel ASIC3, which is a proton-gated Na^+^ channel that has been implicated in pain transduction associated with acidosis in inflamed or ischemic tissues [30]. However, this peptide also inhibits Na_V_1.2 and Na_V_1.8 channels, and to a lesser extent Na_V_1.6 [31], as well as the human ether-a-go-go-related (hERG) potassium channel (K_V_11.1) [32]. APETx1 was identified initially as a gating modifier of the hERG channel [33], but is also promiscuous, inhibiting mammalian Na_V_1.2–Na_V_1.6 and Na_V_1.8 channels [31]. Recently, a new member of the APETx family, APETx4, was shown to have activity on a potential anti-cancer target, the human ether-à-go-go channel (hEag1 or K_V_10.1), but this peptide also inhibits other K_V_ and Na_V_ channels [34]. The demonstrated lack of target specificity for members of the APETx family limits their application as pharmacological tools, although analogues with specific mutations have the potential to overcome this limitation [31]. Peptides from the sea anemone *Heteractis crispa* (APHC1, APHC2 and APHC3) are active on TRPV1 receptors [35,36]. 

The structures of APETx1, APETx2 and BDS-I (which acts on channels containing K_V_3 subunits, including K_V_3.4 [37], but also modulates Na_V_ channels [38]) are similar to those of the Na^+^-channel toxins such as AP-A although quite distinct from those of the ShK/BgK family of toxins (see below). As noted previously [5], sea anemones use common structural scaffolds to create blockers for distinct targets (AP-A, APETx1 and APETx2 act on VGSC, hERG and ASIC channels, respectively), while also using different scaffolds (all-β in APETx1 vs. all-α in ShK) to block similar channels (hERG and K_V_1, respectively). 

Recent proteomic analyses of the venoms of *Stichodactyla haddoni* [39] and *Aulactinia japonicus* (designated *Cnidopus japonicas* in the title of the paper) [40] identified many new toxin families, a number of them with novel cysteine frameworks. A similar analysis of the mucus of *Heteractis magnifica* revealed the presence of hundreds of peptides [41]. No doubt some of these new peptide families will prove to have useful pharmacological properties and may eventually become therapeutic leads. The next section focuses on a peptide for which this clearly is the case.

## 4. Potassium Channel Blockers from Sea Anemones: Therapeutic Leads for the Treatment of Autoimmune Diseases

The voltage-gated K^+^ channel K_V_1.3 is involved in the activation of a sub-set of *T* lymphocytes known as effector memory *T* (*T*_EM_) cells as it regulates the membrane potential during activation by allowing K^+^ efflux to counterbalance the influx of Ca^2+^ from intracellular stores and through CRAC channels [42]. Blocking K_V_1.3 channels in *T*_EM_ cells blocks their activation and proliferation. As *T*_EM_ cells are key mediators of autoimmune diseases, K_V_1.3 blockers are attractive leads as a new class of therapeutic for these conditions [43,44]. 

**Figure 4:**
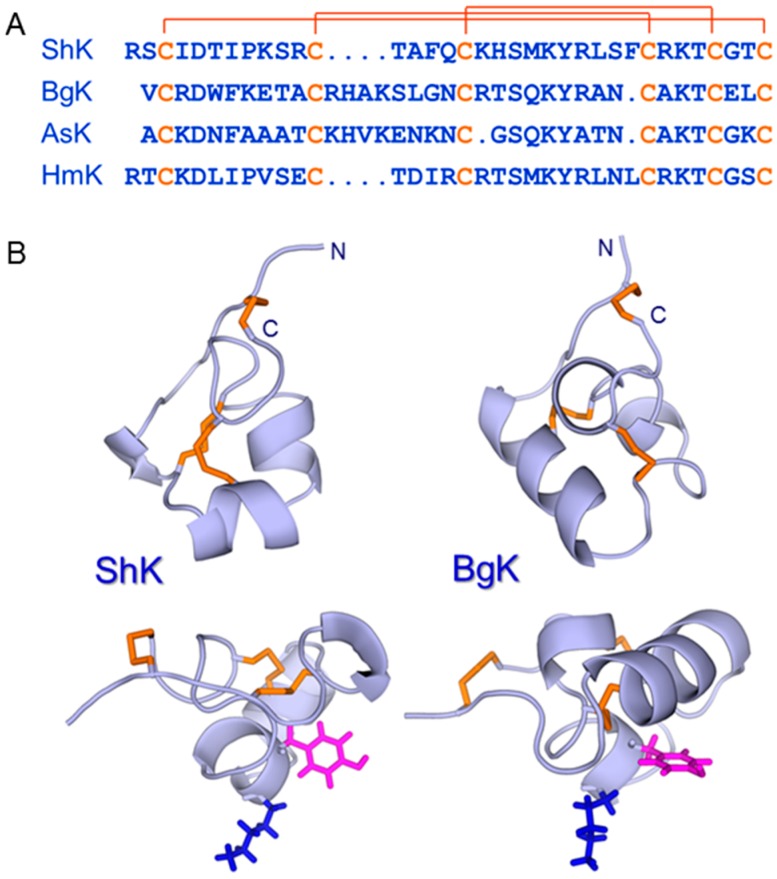
(**A**) Amino acid sequences of ShK (UniProt entry P29187) [48], BgK (P29186) [65], AsK (Q9TWG1), also known as kaliseptine [66], and HmK (O16846) [67] with the three disulfide bonds indicated. (**B**) Structures of ShK (pdb id 1ROO) [50] and BgK (pdb id 1BGK) [51] are shown, with backbones in lightblue and disulfides in orange. The side chains of the Lys and Tyr that constitute the functional dyad [51] in each peptide are shown in blue and magenta, respectively. This dyad is displayed on a helical scaffold in ShK but a β-sheet in the scorpion toxins charybdotoxin and margatoxin (not shown).

Several peptide toxins from scorpions were found to be potent blockers of K_V_1.3 [45,46], but did not progress to clinical trials for a variety of reasons, including the lack of desired specificity for this channel over related K_V_1 channels [47]. Around the same time, a novel peptide, ShK, was isolated from the Caribbean sun anemone, *Stichodactyla helianthus* [48]. This 35-residue peptide was found to be a potent competitive inhibitor of α-dendrotoxin binding to rat brain synaptosomes and blocked K^+^ current in dorsal root ganglion cells. Its amino acid sequence [48] (Figure 4), disulfide bonding pattern [49] and solution structure [50] were all very different from the scorpion toxins, although Lys22 and Tyr23 in ShK, the two key residues for K_V_1.3 blockade, are spatially conserved in an arrangement common to K_V_-channel blocking peptides from widely different species [51]. ShK has a very high affinity (*K*_i_ ~ 10 pM) for K_V_1.3 channels but also displays high pM affinity for K_V_1.1, K_V_1.4 and K_V_1.6, which are present in brain and cardiac tissues [52,53]. In order for this promising peptide toxin to progress to clinical trials, therefore, more selective analogues had to be developed [54]. This eventually led to ShK-186, which had a 100-fold improvement in selectivity for K_V_1.3 over K_V_1.1, K_V_1.4 and K_V_1.6 [55]. 

ShK and its analogues had already shown efficacy in animal models of human autoimmune diseases such as multiple sclerosis and rheumatoid arthritis [56,57], and preclinical testing of ShK-186 yielded favourable results in both rats and monkeys [58]. Unexpectedly, ShK-186 was found to have a long half-life at the site of sub-cutaneous injection, resulting in sustained high pM levels in plasma and a prolonged therapeutic efficacy [58]. ShK-186, which is now known as dalazatide, completed Phase 1a and 1b trials in 2016. The Phase 1b trial in mild-to-moderate plaque psoriasis patients showed that dalazatide was well tolerated and reduced psoriatic skin lesions [59]. It is expected to begin Phase 2a trails in 2018. Dalazatide is being advanced as a treatment for various autoimmune diseases, including inclusion body myositis, lupus, ANCA vasculitis, multiple sclerosis, psoriasis, psoriatic arthritis, rheumatoid arthritis, type 1 diabetes and inflammatory bowel diseases [44,45]. New analogues of ShK with good selectivity for K_V_1.3 over other K_V_1 channels have also been developed [60,61,62,63,64].

The progress of ShK analogues towards the clinic has stimulated interest in this class of peptides, driven partly by the potential for finding additional members of this family of peptides in sea anemones that might be selective for K_V_1.3 or the many other K_V_ channels that are potential therapeutic targets [45]. As a result, it has become apparent that the ‘ShK fold’, typified by ShK [50] and BgK [51], is widely distributed not only in sea anemones and other cnidarians [68,69,70], but also throughout nature. The next section highlights this broad distribution.

## 5. ShK: A Privileged Scaffold in Nature?

In 2010, the Simple Modular Architecture Research Tool (SMART) database (http://smart.embl-heidelberg.de/) predicted the existence of a large superfamily of proteins that contain domains resembling ShK or BgK, which were referred to collectively as ShKT domains [71]. These domains were distributed across nearly 400 proteins from both the plant and animal kingdoms, including Viridiplantae, *Arabidopsis thaliana*, *Oryza sativa*, and green alga *Ostreococcus* sp.; Protozoa, *Cryptosporidium parvum*; Cnidaria, sea anemones, hydra, and jellyfish; Echinodermata, sea urchin; Mollusca, bivalve clams and oysters; Ciona, sea squirt *Ciona intestinalis*; Actinopterygii, zebrafish *Danio rerio* and pufferfish *Takifugu rubripe*s; Caenorhabditis, *C. elegans* and *C. brigssae*; Rhabditida, rhabditid nematodes other than *Caenorhabditis* sp.; Ophidia, snakes; Xenopus, *Xenopus tropicalis*; Aves, chicken *Gallus gallus*; Mammalia. Many of these proteins (~70) were metallopeptidases, whereas others were prolyl-4-hydroxylases, tyrosinases, peroxidases, oxidoreductases, or proteins containing epidermal growth factor-like domains, thrombospondin-type repeats, or trypsin-like serine protease domains [71].

The only human protein containing a ShKT domain in the SMART data base was matrix metalloprotease 23 (MMP23) [72,73]. A second human protein with an ShKT domain, microfibril associated protein MFAP2, was not mentioned in the SMART database at that time [74]. The cysteine-rich secretory proteins (Crisp), which are found predominantly in the mammalian male reproductive tract as well as in the venom of reptiles, are two-domain proteins, one domain of which is an ShKT domain. For example, murine Tpx-1 (testis specific protein-1) contains two subdomains, one of which has a similar fold to BgK and ShK [75]. The Tpx-1 Crisp domain inhibited the cardiac ryanodine receptor (RyR2) and activated the skeletal RyR1.

By 2014, the SMART database identified 668 proteins that contained 1315 ShKT domains. The largest family was found in worms, with 276 of those 668 proteins coming from *Caenorhabditis elegans*, *C. briggsae*, *Brugia malayi*, *B. pahangi*, *Ancylostoma ceylanicum*, *Schistosoma mansoni* and *Toxocara canis* [76]. This prompted an investigation of whether worm ShKTs share structural similarity to ShK, block K_V_1.3, and exhibit immunomodulatory activity. Based on phylogenetic analysis, two worm peptides were selected for study: AcK1, a 51-residue peptide expressed in the anterior secretory glands of both the dog-infecting hookworm *Ancylostoma caninum* and the human-infecting hookworm *Ancylostoma ceylanicum*, and BmK1, the C-terminal domain of a metalloprotease from the filarial worm *Brugia malayi*. These peptides proved to have helical structures closely resembling that of ShK [76]. They also blocked K_V_1.3 channels, selectively suppressed the function of *T*_EM_ lymphocytes, and inhibited delayed-type hypersensitivity. It was suggested that ShK-like worm peptides may be among the active principles that contribute to the well-known protective effect of parasitic worms in autoimmune diseases [76], although further work is required to confirm this hypothesis.

Recently, we have investigated several new ShK-like peptides identified in anemone transcriptomes. One such peptide is AsK132958, from *Anemonia sulcata*, which had an ShKT cysteine framework but was six amino acid residues shorter [77]. The disulfide connectivities and structural scaffold were very similar to those of ShK, although the structure was more constrained. However, AsK132958 showed no activity against grass shrimp, *Artemia nauplii*, or any of the K_V_ channels tested, owing partly to the absence of a functional Lys-Tyr dyad. It appears that Lys19, which would be expected to occupy the pore of the channel, was not sufficiently accessible for binding, and therefore that AsK132958 must have a distinct functional role that does not involve K_V_ channels. The evolutionary relationship between AsK132958 and other ShK-like amino acid sequences is unclear. AsK132958 may represent the shortest peptide sequence that can support a stable ShKT fold, although this remains to be confirmed.

Another peptide, identified in the transcriptome of an *Oulactis* species, was similar to BgK in terms of amino acid sequence and three-dimensional structure, and furthermore contained a Lys-Tyr dyad, but was inactive against K_V_ channels tested to date (our unpublished results). These findings highlight the likely diversity of functions supported by this versatile scaffold.

At the time of writing, the SMART database includes 3345 ShKT domains spread across 1797 proteins, a huge increase over the numbers documented in 2010 [71] and 2014 [76]. These domains are found mainly in animals and plants, but also occur in fungi, viruses and undefined kingdoms. With the dramatic expansion in the number of genomes and transcriptomes spanning all kingdoms, this number is set to increase rapidly. The next section discusses these developments in more detail and outlines how we might begin to assess the functions of these domains.

## 6. New Methods for the Large-Scale Detection of Known and Novel Peptide Toxins

The large-scale identification of new peptide toxins, as well as the evolutionary analysis of known peptide toxins, in sea anemones has been limited by a lack of genomic resources for many species. This is changing rapidly, however, as transcriptome and genome assemblies are being generated using next generation sequencing platforms for a number of sea anemone species [39,78,79,80]. Currently, the genome sequences of *Nematostella vectensis* [81] and *Exaiptasia pallida* are publicly available, as well as transcriptomes of ~30 anemone species from a range of anemone superfamilies. These genomic resources have enabled the in-depth characterization of gene families in sea anemones, including immune gene families [82], and some detailed investigations of select toxin gene families (e.g., actinoporins, which are cytolytic proteins [83]). These genomic resources can be used to study the number, distribution and evolution of a range of candidate toxin gene families in anemones. For example, we were able to identify 71 and 90 non-redundant open reading frames with ShKT domains from previously-published, high-quality transcriptome assemblies for *Calliactis polypus* and *Actinia tenebrosa* [82], respectively (our unpublished results). From these peptide sequences, we were able to identify canonical ShK (Type I KTxs) toxin peptides with a single domain, as well as a many proteins with multiple ShKT domains, and in some instances ShKT domains with other toxin domains. Proteins with multiple ShKT domains have been identified previously in other anemone species, including *Megalactis griffithsi* and *Anemonia sulcata* [79]. Further information about the expression of toxin gene families can be derived by application of state-of-the-art transcriptomic based gene expression studies across nematocyst-rich tissues in anemone species. 

**Figure 5:**
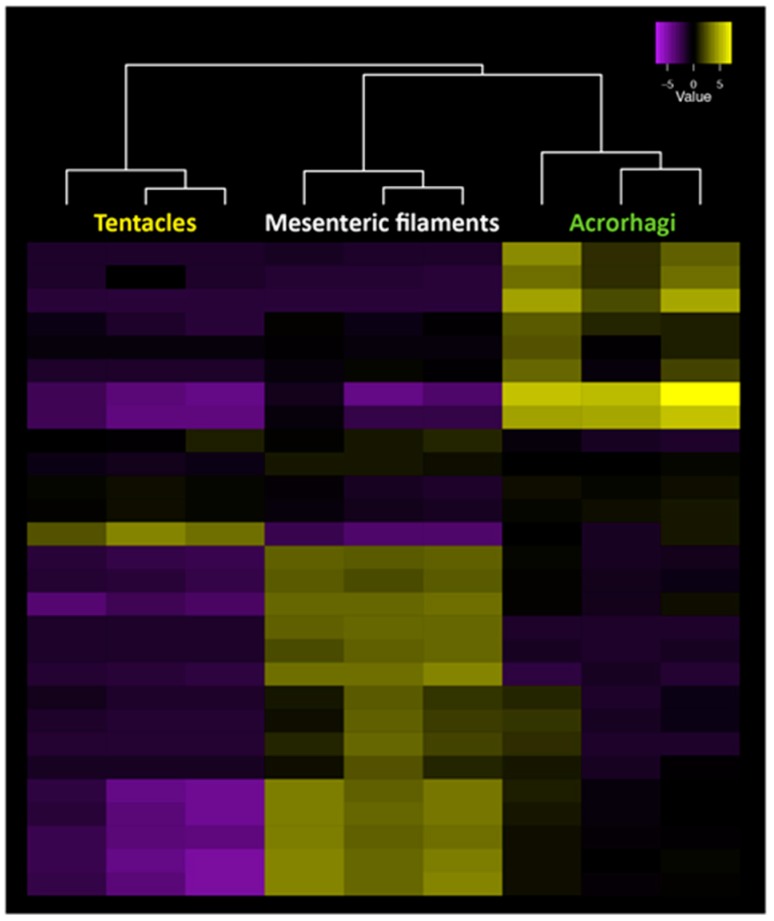
Gene expression patterns for all gene copies of the venom Kunitz gene family across tentacles, mesenteric filaments and acrorhagi in *A. tenebrosa*. Tissue-specific expression can be observed among copies across the three tissues, with significantly upregulated transcripts in yellow and downregulated transcripts in purple (our unpublished data).

Gene expression analysis across morphological structures in sea anemones has shown that many toxin genes are differentially expressed across tissues, presumably resulting in the production of distinct venom combinations in different structures [79]. In fact, specific genes from toxin gene families are expressed precisely in a region-specific pattern, with different gene copies expressed in specific structures, such as tentacles, acrorhagi or mesenteric filaments, as exemplified for the venom Kunitz peptides in Figure 5. This highlights that different genes from known toxin gene families show strong regionalization in their expression patterns across nematocyst-rich tissues in a pattern consistent with changes in nematocyst morphology and density (our unpublished results). Testing whether different nematocyte populations are contributing directly to the changes in toxin gene expression across tissues will require an examination of single-cell gene expression changes in nematocyte populations isolated from specific tissues. The analysis of gene expression profiles from single cell populations has improved greatly over recent years [84] and has led to a precise understanding of the function of individual cell types in a range of species [85]. Determining single-cell venom gene expression patterns in nematocyte populations will provide unprecedented insight into the evolution of venom composition and how functionally different venoms can be produced by a single animal. While this information is useful in understanding the evolution and composition of venoms, the identification of peptide toxins with new modes of action will require proteomic analyses of the venom.

High-throughput proteomic analyses of sea anemone venoms have been greatly accelerated through the use of mass spectrometry [86] coupled with the availability of complete genome and transcriptome sequences for sea anemones species. MALDI imaging [87,88] also offers the prospect of mapping the tissue distributions of numerous peptides, and thus providing a clue to their possible function in the anemone. Recent proteomic research has assayed venom composition in the nematocytes of a few anemone species [69,86], revealing a high frequency of novel peptides and proteins in nematocysts, and in some cases detecting peptides that had not been identified in earlier proteomics studies [89,90,91]. Many of these represent novel candidate toxins and show little overlap among species, highlighting the potential for proteogenomic approaches to identify previously uncharacterized toxins. Another recent study used a combination of proteomic and transcriptomic techniques to analyse the protein composition of milked venom from the sea anemone *Stichodactyla haddoni* [39]; the milked venom contained 23 putative toxin families, 12 of which showed no identity to any known peptide or protein in current databases. The identification of these 12 cysteine-rich, but previously unknown, putative toxins further emphasizes that a proteogenomic approach can greatly accelerate the discovery of novel proteins with potential therapeutic applications.

Continual improvements in the efficiency of peptide synthesis and recombinant expression will facilitate the production of such peptides for biological evaluation, although the challenge will remain to define the native disulfide connectivities for novel sequences containing multiple cysteine residues. New peptides need to be assessed against a range of targets, and in multiple functional assays, in order to identify those with the potential to be new therapeutic leads or at least pharmacological tools. For the most part, these assays will focus on mammalian targets, leaving open the intriguing but often neglected question of what function(s) these peptides have in the anemones in which they are identified. As noted above, MALDI imaging studies of peptide distribution in the anemone should assist in identifying their endogenous functions.

It is to be hoped that new bioinformatic approaches, such as weighted gene co-expression network analysis, may provide preliminary information on the function of candidate peptides and proteins. Weighted gene co-expression analysis categorizes genes into groups whose expression levels are highly correlated across samples [92]. This approach has been used to identify gene clusters with functions in development and lipid metabolism, among others [93,94]. In sea anemones, identifying gene clusters with correlated expression levels that contain known toxins may offer a way to detect novel toxin genes with similar or novel functions.

## 7. Conclusions

Sea anemones remain relatively under-explored as a source of peptide toxins, although that is beginning to change as the transcriptomes and genomes of several species from different geographic regions and different habitats are being investigated. Proteomics studies on sea anemones [39] are more challenging than for other venomous species, where the venom apparatus can be readily milked or dissected, but ongoing enhancements in the resolution and sensitivity of liquid chromatography-mass spectrometry methods will enable characterization of the complete peptide repertoires of even limited quantities of venom when it can be obtained.

The progress of an analogue of ShK through clinical trials for autoimmune diseases emphasizes the promise of this scaffold and, more broadly, of sea anemone peptides. The abundance of ShKT domains in nature and their distribution across different phyla pose questions about the diversity of functions supported by this scaffold. Defining the relationships among sequence, structure and function remains one of the exciting challenges of ongoing studies.

## Figures and Tables

**Table 1:** Characterized toxin protein families identified in sea anemone species in the current ToxProt database, and the number of proteins from each toxin protein family identified in each of the sea anemone superfamilies. Act—Actinioidea; Edw—Edwardsioidea; Met—Metridioidea; Acti—Actinernoidea.

Toxin Protein Family	Anemone Superfamily			
	Act	Edw	Met	Acti
Actinoporin family, Sea anemone subfamily	25	0	5	0
Cnidaria small cysteine-rich protein (SCRiP) family	2	0	1	0
Peptidase M12A family	0	1	0	0
Phospholipase A2 family	3	0	1	0
Sea anemone 8 toxin family	5	0	0	0
Sea anemone short toxin (type III) family	8	0	0	0
Sea anemone sodium channel inhibitory toxin family	0	0	3	0
Sea anemone sodium channel inhibitory toxin family, Type I subfamily	52	0	0	0
Sea anemone sodium channel inhibitory toxin family, Type II subfamily	13	9	0	1
Sea anemone structural class 9a family	6	0	0	0
Sea anemone type 1 potassium channel toxin family, Type 1a subfamily	9	0	0	0
Sea anemone type 1 potassium channel toxin family, Type 1b subfamily	11	0	1	0
Sea anemone type 3 (BDS) potassium channel toxin family	32	0	0	0
Sea anemone type 5 potassium channel toxin family	1	1	1	0
Venom Kunitz-type family, Sea anemone type 2 potassium channel toxin subfamily	26	0	0	0
Unknown	15	0	4	0
